# Evidenzlage zu Advance Care Planning bei Menschen mit Demenz – Ein systematisches Review (2017–2023)

**DOI:** 10.1055/a-2699-8545

**Published:** 2025-11-27

**Authors:** Jana Rühl, Alina Baumgartner, Peter Kolominsky-Rabas

**Affiliations:** 1 Friedrich-Alexander-Universität Erlangen-Nürnberg, Interdisziplinäres Zentrum für Health Technology Assessment (HTA) und Public Health (IZPH), Erlangen, Germany

**Keywords:** Demenz, Advance Care Planning, Vorausschauende Versorgungsplanung, pflegende Angehörige, dementia, Advance Care Planning, advance directive, caregiver

## Abstract

**Hintergrund:**

Für Menschen mit Demenz ist eine frühzeitige Auseinandersetzung mit Themen der Versorgungsplanung von zentraler Bedeutung. Das Konzept des Advance Care Planning (ACP) bietet eine vielversprechende Möglichkeit ihre individuellen Wünsche in Hinblick auf Pflege und Behandlung am Lebensende zu berücksichtigen. Ziel Ziel dieser Arbeit ist es eine Übersicht zur aktuellen Evidenzlage zum Thema Advance Care Planning bei Menschen mit Demenz bereitzustellen.

**Methodik:**

Es wurde eine systematische Literaturrecherche in den internationalen Datenbanken Medline, PsycINFO, Scopus, CINAHL und CENTRAL für den Zeitraum von Januar 2017 bis April 2023 durchgeführt. Die Studienqualität der eingeschlossenen Studien wurde mittels Critical Appraisal Skills Programme (CASP) bewertet.

**Ergebnisse:**

Sieben Studien konnten in der qualitativen Analyse berücksichtigt werden. Die Studien zeigen positive Effekte des Advance Care Planning auf unterschiedliche Domänen für Menschen mit Demenz und ihre Pflegenden. Die eingeschlossenen RCTs weisen allerdings große Heterogenität hinsichtlich Methodik, Qualität und ihrer erhobenen Endpunkte auf.

**Schlussfolgrung:**

Videos und Schulungen können ein wirksames Instrument sein, um ACP für Menschen mit Demenz und ihre Pflegenden erfolgreich zu implementieren. Allerdings sind Studien mit strengen Interventionskriterien und einheitlichen Endpunkten erforderlich, um die Wirksamkeit zu Advance Care Planning abschließend beurteilen zu können.

## Abstract (en)

**Background:**

Early involvement in care planning is essential for people with dementia. The concept of advance care planning (ACP) offers a promising opportunity to consider their individual wishes for care and treatment at the end of life. Aim The aim of this review was to provide an overview of the current evidence on advance care planning for people with dementia.

**Methods:**

We conducted a systematic literature search in the databases Medline, PsycINFO, Scopus, CINAHL, and CENTRAL for articles published between January 2017 and April 2023. The study quality of the included studies was assessed using the Critical Appraisal Skills Programme (CASP).

**Results:**

Seven studies were included in the qualitative analysis. The studies demonstrated positive effects of Advance Care Planning on various domains for individuals with dementia and their caregivers. However, the included RCTs exhibited significant heterogeneity in terms of methodology, quality, and measured outcomes.

**Conclusion:**

Videos and training can be effective tools for successfully implementing ACP for individuals with dementia and their caregivers. However, studies with stringent intervention criteria and standardized outcomes are needed to conclusively assess the effectiveness of Advance Care Planning.

## Hintergrund

Demenzerkrankungen und ihre Folgen sind eine der größten gesellschaftlichen Herausforderungen unserer Zeit. Global betrachtet waren im Jahr 2019 57,4 Millionen Menschen betroffen. Aktuelle Prognosen gehen bis zum Jahr 2050 sogar von einem Anstieg auf 152,8 Millionen aus 1 . Trotz erster Hinweise auf positive Auswirkungen von krankheitsmodifizierenden Behandlungen und Lebensstilinterventionen 2 , handelt es sich bei einer Demenz in der Regel um eine lebenslimitierende Erkrankung 3 . Einer aktuellen Übersichtsarbeit zufolge beträgt die Überlebenszeit nach Einsetzen erster Symptome im Durchschnitt 7,6 Jahre 4 . Die Dauer kann jedoch abhängig von verschiedenen Faktoren, wie Alter, Geschlecht und Bildungsstand sowie dem Schweregrad der Erkrankung oder dem Auftreten spezifischer Symptome, stark variieren 4 5 . Todesursächlich sind häufig kardiovaskuläre und respiratorische Komplikationen, wie zum Beispiel Pneumonien, ischämische Herzerkrankungen oder Herzinfarkte 6 7 8 , die mit der Demenzerkrankung einhergehen. Im fortgeschrittenen Stadium ist die Erkrankung geprägt von schweren kognitiven sowie körperlichen und neurologischen Beeinträchtigungen 3 9 . Darüber hinaus treten vermehrt verhaltensbezogene und psychologische Symptome auf. Folglich nimmt auch der Unterstützungsbedarf im Alltag und der Pflegebedarf Betroffenen zu.

Damit Menschen mit Demenz (MmD) noch aktiv in die Behandlungsplanung miteinbezogen werden können, ist vor dem Hintergrund der Progredienz der Erkrankung, eine rechtzeitige Auseinandersetzung mit den Wünschen und Bedürfnissen der Betroffenen von besonderer Bedeutung. 10 11 . Eine vielversprechende Möglichkeit, um die Präferenzen für die künftige medizinische Versorgung im Vorfeld strukturiert zu eruieren und zu dokumentieren, ist das Konzept des Advance Care Planning (ACP) 10 11 12 . ACP dient dabei der Realisierung wirksamer Patientenverfügungen und Vorsorgevollmachten 13 . Entstanden ist dieser Ansatz in den 1990er Jahren in den USA 14 und hat in der Zwischenzeit sowohl international als auch in Deutschland zunehmend an Bedeutung gewonnen 15 . Während in Literatur und Praxis eine Vielzahl unterschiedlicher Definitionen und Modelle von ACP existiert 12 16 17 , orientieren sich die Autoren im Rahmen dieses systematischen Reviews an den Grundsätzen der in einem internationalen Delphi-Verfahren entwickelten Konsensusdefinition von ACP 12 . Demnach ist ACP „ein Prozess, der Erwachsene in jedem Alter oder Gesundheitszustand dabei unterstützt, ihre persönlichen Werte, Lebensziele und Präferenzen in Bezug auf die künftige medizinische Versorgung zu verstehen und mitzuteilen“ 12 und dessen Ziel es ist, „dazu beizutragen, dass Menschen bei schweren und chronischen Erkrankungen eine medizinische Versorgung erhalten, die mit ihren Werten, Zielen und Präferenzen übereinstimmt“ 17 .

Bereits in der Vergangenheit haben sich einige systematische Übersichtsarbeiten mit ACP bei MmD befasst und kamen zu heterogenen Ergebnissen 10 17 18 19 . Dieses systematische Review verfolgt daher das Ziel an die bereits vorhandenen Erkenntnisse anzuknüpfen und die aktuelle Evidenzlage zur Wirksamkeit von ACP-Interventionen zu untersuchen. Um möglichst zuverlässige Informationen zur Wirksamkeit von ACP bei MmD und ihren Pflegenden zu gewinnen, wurden insbesondere hinsichtlich des Studiendesigns strengere Einschlusskriterien für die Studien gewählt als in vorangehenden Übersichtsarbeiten.

## Methodik

Zur Erfassung der Studienlage wurde ein systematisches Review durchgeführt. Das methodische Vorgehen folgte den Standards des „Preferred Reporting Items for Systematic reviews and Meta-Analyses“ (PRISMA) 20 und wurde vorab im „National prospective register of systematic reviews“ (PROSPERO) (Registrierungsnummer: CRD42021277558) registriert. Für die Präzisierung der Fragestellung wurde das PICO-Schema herangezogen.

### Suchstrategie

Die systematische Literaturrecherche fand in den Datenbanken CINAHL, CENTRAL, MEDLINE, PsycINFO und Scopus statt. Zusätzlich wurden die Literaturangaben relevanter Artikel auf weitere geeignete Referenzen durchsucht. Die Suchmatrix bestand aus Begriffen zu Demenz und ACP. Sofern möglich, wurde die Suche mit Filtern zu Sprache und Publikationszeitraum präzisiert (Online Supplement 1).

### Ein- und Ausschlusskriterien

Eingeschlossen wurden alle randomisiert kontrollierte Studien (RCTs), die zwischen Januar 2017 und April 2023 in deutscher oder englischer Sprache publiziert wurden. Zielgruppe der Intervention waren MmD, ihre pflegenden An- und Zugehörigen (pA) oder medizinisches Fachpersonal (Fp). Gemäß der Konsensusdefinition von Sudore et al. 12 sollte die Intervention einen Prozess zur Ermittlung und Dokumentation der individuellen Werte und Präferenzen in Hinblick auf die künftige medizinische Versorgung umfassen. In Anlehnung an die Erkenntnisse aus der Übersichtsarbeit von Wendrich-van Dael et al. 17 konnte es sich dabei um einen mehrstufigen, freiwilligen, interaktiven, kontinuierlichen und formalisierten Prozess handeln. Beinhalten konnte dieser eine Diskussion oder ein Gespräch über Ziele und Präferenzen für die künftige Versorgung zwischen Einzelpersonen und medizinischem Fachpersonal, oder zwischen Betroffenen und informellen Pflegepersonen oder zwischen Patienten, informeller Pflegperson und medizinischem Fachpersonal. Die Kontrollgruppen sollten die Standardversorgung oder alternative Interventionen ohne ACP erhalten. Aufgrund der mangelnden Standardisierung und des breiten Spektrums an Endpunkten, die zur Bewertung von ACP-Interventionen verwendet werden, wurden keine festen Endpunkte festgelegt. Alle Endpunkte, die die Wirksamkeit von ACP erfassten, wurden eingeschlossen (Online Supplement 2).

### Studienauswahl und -qualitätsbewertung

Gemäß der PRISMA-Kriterien selektierten zwei unabhängige Begutachterinnen (AB, JR) die Titel und Abstracts nach den definierten Ein- und Ausschlusskriterien. Bei unklarer Einschlusseignung wurde Einigung durch Diskussion mit einem dritten Gutachter (PK) erzielt. Alle relevanten Informationen, wie Studien-, Interventions- und Probandencharakteristika sowie Outcomes wurden aus den Artikeln in standardisierte Tabellen extrahiert. Die Qualität der Studien wurde mittels Critical Appraisal Skills Programme (CASP) für RCTs 21 beurteilt. Die Checkliste umfasst 11 Fragen zu Stringenz, Forschungsmethode, Relevanz und Forschungsintegrität. Die Studienbewertungen wurden von zwei unabhängigen Reviewerinnen (AB, JR) durchgeführt. Diskrepanzen wurden durch Diskussion ausgeräumt.

## Ergebnisse

### Eingeschlossene Studien

**Abb. 1:**
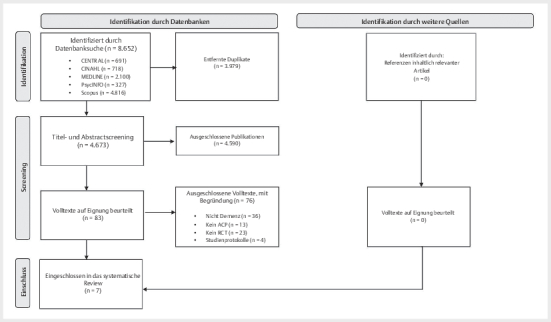
PRISMA-Flussdiagramm der Literaturrecherche. (Moher D, Liberati A, Tetzlaff J, Altman DG, The PRISMA Group (2009). Preferred Reporting Items for Systematic Reviews and Meta-Analyses: The PRISMA Statement. PLoS Med 6(7): e1000097. doi:10.1371/journal.pmed1000097).

Insgesamt konnten im Rahmen der systematischen Literaturrecherche 8.652 Studien identifiziert werden. Nach Entfernung von Duplikaten und Screening von Titeln und Abstracts wurden 83 Studien anhand ihrer Volltexte beurteilt. Sieben dieser Studien wurden schlussendlich in der Synthese berücksichtigt 22 23 24 25 26 27 28 ( Abb. 1 ).

Bei allen eingeschlossenen Studien handelte es sich um clusterrandomisierte, kontrollierte Studien 22 23 24 25 26 27 28 . In zwei Studien wurden die Teilnehmenden zu ACP bei moderater bis schwerer Demenz geschult 24 26 . Eine Studie fokussierte sich auf Menschen mit schwerer Demenz 23 . In zwei Fällen wurden alle Schweregrade berücksichtigt 27 28 , wohingegen in zwei Studien keine näheren Angaben dazu gemacht wurden 22 25 . Die Mehrheit der Studien wurde in Pflegeheimen durchgeführt 23 25 26 27 . Eine Studie wurde mithilfe von Allgemeinärzten unterschiedlicher Praxen umgesetzt 28 , eine fand im Kontext afroamerikanischer, religiöser Gruppen statt 24 und eine im Rahmen ambulant betreuter Wohneinrichtungen 22 . Bei den Interventionen handelte es sich um Schulungen, Trainingsseminare oder Workshops 22 24 25 28 , Videos 23 26 und Meetings von pA mit einer geschulten Pflegefachkraft 27 ( Tab. 1 ).

**Table:** **Tab. 1** Studiencharakteristika

Autor, Jahr	Land	ZG	IG	KG	Setting	Demenz-schweregrad	Intervention	Kontrolle	ID/FU-Zeitpunkte
Bonner et al. 2021	USA	pA	n=173 pA	n=185 pA	4 afro-amerikanische Kirchen	moderat bis schwer	Unterrichtseinheiten, Hausaufgaben, interaktive Diskussionen zu ACP	Unterrichtseinheiten, Hausaufgaben, interaktive Diskussionen zu Gesundheitsförderung	ID: 4 W, wöchentlich je 1 Std FU: 4, u. 20 W nach Intervention
Brazil et al. 2018	Nordirland	pA	n=67 pA	n=117 pA	24 Pflegeheime	alle	Meetings mit geschulter Pflegekraft, ACP-Handbuch	Standardpflege	ID: 2 Meetings FU: 6 W nach Baseline-Erhebung; Überprüfung der Patientenakten 6 M nach FU-Erhebung
Dobbs et al. 2023	USA	Fp	n=17 pP n=73 MmD	n=6 pP n=45 MmD	10 betreute Wohn-einrichtungen	nicht berichtet	ACP-Unterrichtseinheiten, Handbuch	Standardpflege	ID: 4 Sessions je 1,5 Std FU: 3 u. 6 M nach Intervention
Goosens et al. 2020	Belgien	Fp	n=160 pP	n=151 pP	46 Pflegeheime	nicht berichtet	ACP-Workshops, Hausaufgaben, Unterstützung bei der Implementierung	kein Training	ID: 1 M, 2 Workshops von je 4 Std FU: 3 u. 6 M nach Intervention
Hanson et al. 2017	USA	pA	n=151 Dyade MmD/pA	n=151 Dyade MmD/pA	22 Pflegeheime	moderat bis schwer	ACP-Video, strukturierte Diskussion mit dem Pflegepersonal	Video zur Interaktion mit MmD, übliches Pflegeplanungstreffen	ID: 18 Min Video und Diskussion FU: 3, 6 und 9 M nach Intervention
Mitchell et al. 2018	USA	pA	n=212 Dyade MmD/pA	n=190 Dyade MmD/pA	64 Pflegeheime	schwer	ACP-Video, Formular zur Dokumentation	übliche ACP-Praktiken	ID: 12 Min Video FU: 3, 6, 9, u. 12 M nach Intervention
Tilburgs et al. 2020	Niederlande	Fp	n=19 pP n=73 Dyade MmD/pA	n=19 pP n=67 Dyade MmD/pA	38 Allgemeinmediziner	alle	ACP-Trainingsprogramm, Handbuch, Telefonberatungen	Informationen über Hintergrund, der Studie, Standardpflege	ID: 2 Workshops je 3 Std FU: 6 M nach Intervention

Abkürzungen: ACP: Advance Care Planning; Fp: Fachpersonal; FU: Follow-Up; ID: Interventionsdauer; IG: Interventionsgruppe; KG: Kontrollgruppe; M: Monate; Min: Minuten; MmD: Menschen mit Demenz; pA: pflegende A- und Zungehörige; Std: Stunden; W: Wochen; ZG: Zielgruppe

### Studienqualität der eingeschlossenen Studien

Die gemäß CASP durchgeführte Bewertung zeigte eine moderate Studienqualität ( Tab. 2 ). Das größte Verzerrungsrisiko lag in der mangelnden Verblindung der Studienteilnehmenden und des Studienpersonals. Der Prozess der Randomisierung konnte mit Ausnahme von einer Studie 24 in allen Fällen nachvollzogen werden. Allerdings zeigten sich in drei Studien 23 24 25 dennoch gewisse Unterschiede in den Zusammensetzungen der Vergleichsgruppen, sodass hier ein gewisses Verzerrungspotential nicht auszuschließen war. Außerdem bestanden aufgrund der relativ kleinen Stichproben (n=118–402) Bedenken hinsichtlich der Generalisierbarkeit der Ergebnisse. In einer Studie wurden zudem keine Angaben zur Signifikanz der Ergebnisse gemacht 22 .

**Table:** **Tab. 2** Beurteilung der Studienqualität nach Critical Appraisal Skills Programme (CASP)

Studie	Hat die Studie eine klar umrissene Fragestellung?	War die Zuweisung der Patienten randomisiert?	Wurden alle Teilnehmenden am Ende der Studie ordnungsgemäß erfasst?	Wurden Patienten, medizinisches Personal und Studien-personal verblindet?	Waren die Gruppen zu Beginn der Studie ähnlich?	Wurden die Gruppen abgesehen von der Intervention gleich-behandelt?	Wie groß war der Effekt der Intervention?	Wie genau war die Schätzung des Behandlungs-effekts?	Können die Ergebnisse auf die lokale Bevölkerung oder auf Ihren Kontext übertragen werden?	Wurden alle klinisch relevanten Outcomes berücksichtigt?	Sind die Vorteile die Schäden und Kosten wert?
Bonner et al. 2021	⊕	⊖	⊕	⊖	⊖	⍰	⊕	⊕	⊖	⊕	⊕
Brazil et al. 2018	⊕	⊕	⊕	⊖	⊕	⍰	⊕	⊕	⊖	⊕	⊕
Dobbs et al. 2023	⊕	⊕	⊕	⊖	⊕	⍰	⊕	⊖	⊖	⊖	⊕
Goosens et al. 2020	⊕	⊕	⊕	⊖	⊖	⍰	⊕	⊕	⊖	⊖	⊕
Hanson et al. 2017	⊕	⊕	⊕	⊖	⊕	⊕	⊕	⊕	⊖	⊕	⊕
Mitchell et al. 2018	⊕	⊕	⊕	⊖	⊖	⍰	⊕	⊕	⊖	⊕	⊕
Tilburgs et al. 2020	⊕	⊕	⊕	⊖	⊕	⍰	⊕	⊕	⊖	⊕	⊕

⊕ ja ⊖ nein ⍰ unklar

### ACP-Interventionen für pflegende An- und Zugehörige

Alle vier eingeschlossenen Studien, die sich an die pA von MmD richteten, konnten positive Effekte der ACP-Interventionen nachweisen 23 24 26 27 ( Tab. 3 ). Dementsprechend verbesserte sich in der IG einer Studie das Wissen zu Demenz und zu lebensverlängernden Maßnahmen sowie die Selbstwirksamkeit der pA der IG signifikant 24 . In einer anderen Studie führte die Intervention zu einer Verringerung des Entscheidungskonflikt der pA und zu einer positiveren Beurteilung der Versorgungssituation, insbesondere hinsichtlich der Unterstützung bei der Entscheidungsfindung und der Kommunikation 27 . Auch die pA der IG der Studie von Hanson et al. erlebten eine signifikant verbesserte Kommunikation. Sie stimmten nach 9 Monaten signifikant öfter mit dem medizinischen Fachpersonal bezüglich der Behandlungsziele überein als die pA der KG und es wurden mehr Behandlungsziele und Patientenverfügungen dokumentiert 26 . Die Videointervention von Mitchell et al. bewirkte ebenfalls mehr Behandlungsziel-Diskussionen und Sondenernährungsverfügungen 23 .

**Table:** **Tab. 3** Ergebnisse für Intervention für pflegende An- und Zugehörige

Studie	Endpunkte	Zeitpunkt		IG	KG	Signifikanz
**Bonner et al. 2021**	Wissen Demenz	4 Wochen		5,18±2,34	2,88±2,46	p=0,1759
		20 Wochen		**10,61±2,51**	**5,48±2,51**	**p=0,0004**
	Wissen CPR	4 Wochen		**15,67±3,60**	**− 1,75±3,75**	**p<0,0001**
		20 Wochen		**14,40±3,85**	**5,81±3,85**	**p=0,0017**
	Wissen MV	4 Wochen		**12,27±3,45**	**2,10±3,61**	**p<0,0001**
		20 Wochen		**15,90±3,70**	**− 0,81±3,69**	**p<0,0001**
	Wissen TF	4 Wochen		**9,69±3,83**	**0,16±4,01**	**p=0,0006**
		20 Wochen		**13,88±4,09**	**− 4,83±4,08**	**p<0,0001**
	Wissen Gesundheitsförderung	4 Wochen		**− 0,75±2,99**	**4,48±3,08**	**p=0,0151**
		20 Wochen		**− 0,02±3,19**	**6,44±3,16**	**p=0,0042**
	Selbstwirksamkeit	4 Wochen		**0,82±0,15**	**0,27±0,16**	**p<0,0001**
		20 Wochen		**0,70±0,16**	**0,26±0,16**	**p<0,0001**
	Absicht	4 Wochen		72,7%±8,3%	68,1%±8,7%	p=0,5474
		20 Wochen		64,1%±10,1%	57,1%±10,3%	p=0,3401
	Verhalten	4 Wochen		13,9%±6,2%	12,9%±6,3%	p=0,8307
		20 Wochen		10,7%±6,0%	14,0%±6,8%	p=0,4722
**Brazil et al. 2018**	Entscheidungskonflikte pA (DCS) Gesamt	Baseline		28,3±22,3	34,7±21,0	
		6 Wochen		**18,3±19,7**	**30,7±20,5**	**p<0,001**
	DCS Informiertheit	Baseline		33,8±26,0	39,5±26,2	
		6 Wochen		**20,2±22,7**	**37,4±25,7**	**p<0,001**
	DCS Werteklarheit	Baseline		33,2±28,3	36,2±24,8	
		6 Wochen		**21,2±25,2**	**32,5±24,0**	**p=0,03**
	DCS Unterstützung	Baseline		26,8±24,2	31,6±21,5	
		6 Wochen		**17,1±19,5**	**27,4 20,9**	**p<0,001**
	DCS Unsicherheit	Baseline		34,4±27,5	38,2±22,2	
		6 Wochen		**21,6±21,6**	**31,8±21,2**	**p=0,01**
	DCS Entscheidung	Baseline		24,4±22,1	29,6±21,7	
		6 Wochen		**16,8±21,0**	**25,8±19,7**	**p<0,001**
	Psychische Belastung pA (GHQ)	Baseline		11,4±5,3	12,6±6,1	
		6 Wochen		9,9±6,1	11,6±5,4	p=0,44
	Beurteilung der Versorgung (FPCS) Gesamt	Baseline		138,0±21,4	131,0±22,9	
		6 Wochen		**144,6±25,6**	**133,6±23,8**	**p=0,01**
	FPCS Versorgung	Baseline		61,1±10,6	59,1±11,3	
		6 Wochen		63,6±12,3	60,1±11,4	p=0,11
	FPCS Familie	Baseline		28,7±7,1	26,8±7,1	
		6 Wochen		**32,7±7,2**	**28,2±7,3**	**p<0,001**
	FPCS Kommunikation	Baseline		34,3±6,9	33,1±5,2	
		6 Wochen		**35,6±6,9**	**33,2±5,4**	**p<0,001**
	FPCS Unterbringung	Baseline		12,9±1,2	12,2±1,8	
		6 Wochen		12,7±1,9	12,2±1,9	p=0,21
	DNR-Verfügungen	6 Wochen		51%	42%	p=0,18
	KH-Einweisungen	6 Wochen		7%	18%	p=0,12
	Sterbeort	PH	6 Wochen	86%	80%	p=0,94
KH		6 Wochen	14%	20%		
**Hanson et al. 2017**	Kommunikationsqualität (QOC) Gesamt	Baseline		5,5±1,7	5,6±1,7	p=0,59
		3 Monate		**6,0±2,0**	**5,6±1,8**	**p=0,05**
		9 Monate		5,8±2,4	5,6±1,9	p=0,19
	QOC Allgemein	Baseline		8,4±1,8	8,4±1,7	p=0,97
		3 Monate		8,6±1,7	8,7±1,6	p=0,63
		9 Monate		**8,2±2,3**	**8,6±1,9**	**p=0,03**
	QOC Lebensende	Baseline		2,9±2,3	3,1±2,3	p=0,53
		3 Monate		**3,7±2,7**	**3,0±2,6**	**p=0,02**
		9 Monate		**3,9±3,1**	**3,1±2,6**	**p=0,03**
	Übereinstimmung Familie mit Gesundheitsdienstleister	Baseline		63,2%	68,2%	p=0,47
		3 Monate		77,2%	70,1%	p=0,12
		9 Monate		**88,4%**	**71,2%**	**p=0,001**
	Komfort als Haupt-BZ	Baseline		63,6%	68,9%	p=0,38
		3 Monate		69,3%	70,3%	p=0,56
		9 Monate		81,0%	78,4%	p=0,55
	ACP Problem-Score≥1	Baseline		53,8%	50,7%	p=0,98
		3 Monate		62,5%	73,9%	p=0,06
		9 Monate		68,3%	66,9%	p=0,82
	Kommunikation über BZ mit Pflege/Sozialdienst	Baseline		89,4%	91,4%	p=0,60
		3 Monate		84,3%	82,6%	p=0,49
		9 Monate		81,0%	81,8%	p=0,93
	Kommunikation über BZ mit Arzt	Baseline		56,3%	55,6%	p=0,78
		3 Monate		25,7%	21,0%	p=0,52
		9 Monate		25,9%	25,0%	p=0,94
	Kommunikation über BZ mit Nurse Practitioner	Baseline		38,4%	36,4%	p=0,55
		3 Monate		30,0%	26,1%	p=0,74
		9 Monate		**36,7%**	**18,9%**	**p=0,02**
	Symptommanagement (SM-EOL)	Baseline		32,7±8,9	33,5±8,5	p=0.56
		6 Monate		32,2±9,0	33,2±8,8	p=0,75
		9 Monate		32,6±9,7	33,7±8,6	p=0,48
	Zufriedenheit mit der Versorgung (SWC-EOLD)	Baseline		30,8±5,4	30,1±5,2	p=0,40
		6 Monate		31,4±5,6	31,7±5,1	p=0,17
		9 Monate		31,0±5,9	31,6±5,3	p=0,16
	Palliativpflege Behandlungsplan Domänen-Score	Baseline		5,1±2,2	4,8±1,9	p=0,26
		6 Monate		**5,6±2,0**	**4,7±1,8**	**p=0,02**
		9 Monate		5,9±2,2	5,3±1,9	p=0,17
	Dokumentierte BZ	Baseline		46%	30%	p=0,06
		6 Monate		**91%**	**42%**	**p<0,001**
		9 Monate		**95%**	**52%**	**p<0,001**
	MOLST-Verfügungen	Baseline		25%	15%	p=0,33
		6 Monate		34%	15%	p=0,12
		9 Monate		**35%**	**16%**	**p=0,05**
	DNR- Verfügungen	Baseline		82%	84%	p=0,97
		6 Monate		84%	90%	p=0,52
		9 Monate		**85%**	**91%**	**p=0,04**
	DNH- Verfügungen	Baseline		23%	23%	p=0,73
		6 Monate		33%	30%	p=0,63
		9 Monate		36%	36%	p=0,91
	DNTF- Verfügungen	Baseline		33%	20%	p=0,22
		6 Monate		45%	26%	p=0,58
		9 Monate		46%	31%	p=0,20
	Symptombehandlungsverfügungen	6 Monate		52%	68%	p=0,71
		9 Monate		67%	75%	p=0,39
	Hospizaufnahmen	Baseline		7	8	p=0,99
		6 Monate		11	13	p=0,72
		9 Monate		17	18	p=0,97
KH-Einweisungen	9 Monate		**0,08**	**0,16**	**p=0,02**
**Mitchell et al. 2018**	Behandlungspräferenz Komfort	Baseline		65,1%	62,1%	[0,85; 1,94]
		3 Monate		72,2%	75,1%	[0,76; 1,94]
		6 Monate		73,2%	76,9%	[0,58; 1,58]
		9 Monate		75,2%	81,0%	[0,38; 1,23]
		12 Monate		76,1%	82,1%	[0,38; 1,38]
	DNH-Verfügungen	3 Monate		60,2%	58,2%	[0,74; 1,77]
		6 Monate		63,0%	63,0%	[0,69; 1,69]
		9 Monate		66,4%	66,1%	[0,66; 1,69]
		12 Monate		68,2%	66,7%	[0,66; 1,72]
	DNTF-Verfügungen	3 Monate		67,3%	57,7%	[0,91; 2,34]
		6 Monate		**70,1%**	**61,9%**	**[1,13; 2,82]**
		9 Monate		**72,0%**	**63,0%**	**[1,22; 2,16]**
		12 Monate		**76,3%**	**64,0%**	**[1,28; 3,91]**
	DNIH-Verfügungen	3 Monate		36,5%	28,0%	[0,91; 2,34]
		6 Monate		37,4%	31,7%	[0,83; 2,12]
		9 Monate		39,8%	34,9%	[0,83; 2,16]
		12 Monate		43,1%	36,0%	[0,93; 2,44]
	BZ-Diskussionen	**3 Monate**		**16,1%**	**7,9%**	**[1,20; 5,54]**
		6 Monate		23,2%	15,3%	[0,94; 3,07]
		9 Monate		29,9%	22,2%	[0,87; 2,54]
		12 Monate		34,1%	25,4%	[0,86; 2,70]

Abkürzungen: ACP: Advance Care Planning; BZ: Behandlungsziel; CPR: cardiopulmonary resuscitation; DNH: Do-Not-Hospitalize; DNIH: Do-Not-Intervenous Hydrate; DNR: Do-Not-Resuscitate; DNT: Do-Not-Tube-Feed; IG: Interventionsgruppe; KG: Kontrollgruppe; KH: Krankenhau; MOLST: Medical Orders for Life-Sustaining Treatment; MV: mechanical ventilation; pA: pflegende An- und Zugehörige; TF: tube feeding

Für eine Vielzahl von Outcomes blieb die Studienlage jedoch uneindeutig und erlaubte keine abschließende Beurteilung der Wirksamkeit von ACP. Während beispielsweise die Ergebnisse von Brazil et al. 27 auf positive Effekte von ACP auf die Zufriedenheit mit der Versorgung hindeuteten, konnten Hanson et al. 26 keine signifikanten Verbesserungen nachweisen. Ebenso lagen keine einheitlichen Ergebnisse hinsichtlich der Auswirkungen von ACP auf die Dokumentation von Pflegezielen und Patientenverfügungen 23 24 26 27 und die Anzahl der Krankenhauseinweisungen 26 27 vor. Signifikante negative Auswirkungen der Interventionen wurden jedoch in keiner der eingeschlossenen Studien berichtet 23 24 26 27 .

### ACP-Interventionen für medizinisches Fachpersonal

In drei der eingeschlossenen Studien war medizinisches Fp die Zielgruppe der ACP-Interventionen 22 25 28 . Hierbei handelte es sich um Mitarbeitende in Pflegeheimen 25 und ambulant betreuten Wohngemeinschaften 22 sowie Allgemeinmediziner 28 . Alle Studien konnten positive Auswirkungen berichten ( Tab. 4 ). So führten die ACP-Interventionen zu einem Anstieg an ACP-Konversationen 22 28 . Für einige Outcomes, wie zum Beispiel die Kompetenz der pA, die Lebensqualität der MmD oder die Gesundheitskosten 28 konnten jedoch keine signifikanten Verbesserungen gegenüber der KG nachgewiesen werden. Hinsichtlich des Einflusses von ACP-Interventionen auf das Niveau der gemeinsamen Entscheidungsfindung zwischen Fp und MmD kamen die eingeschlossenen Studien zu heterogenen Ergebnissen. Während die Werte in der Studie von Goossens et al. 25 durch die Intervention signifikant anstiegen, wurden in der Studie von Tilburgs et al. 28 keine eindeutigen Auswirkungen deutlich.

**Table:** **Tab. 4** Ergebnisse für Interventionen für medizinisches Fachpersonal

Studie	Endpunkte	Zeitpunkt	IG	KG	Signifikanz
**Dobbs et al. 2023**	ACP-Diskussionen	Baseline	12,3%	8,2%	–
		3 Monate	50,1%	12,0%	–
		6 Monate	51,1%	23,0%	–
	Hospizeinweisung	Baseline	0,0%	0,0%	–
		3 Monate	11,7%	3,8%	–
		6 Monate	69,4%	75,0%	–
	Schmerzscreening	Baseline	17,9%	22,3%	–
		3 Monate	31,0%	35,6%	–
		6 Monate	39,0%	47,3%	–
**Goosens et al. 2020**	SDM Level (OPTION-12)	Baseline	26,59±9,36	27,46±11,74	
		3 Monate	**53,49±13,16**	**24,98±9,22**	**p<0,001**
		6 Monate	**56,00±11,57**	**22,27±9,33**	**p<0,001**
	SDM Wichtigkeit (IFC-SDM)	Baseline	4,46±0,42	4,50±0,42	
		3 Monate	**4,64±0,36**	**4,49±0,45**	**p=0,031**
		6 Monate	4,62±0,44	4,56±0,42	p=0,458
	SDM Frequenz (IFC-SDM)	Baseline	3,45±0,86	3,56±0,87	
		3 Monate	3,67±0,88	3,48±0,84	p=0,201
		6 Monate	3,75±0,90	3,59±0,84	p=0,436
	SDM Kompetenz (IFC-SDM)	Baseline	3,73±0,52	3,79±0,48	
		3 Monate	**3,91±0,31**	**3,75±0,38**	**p=0,010**
		6 Monate	**3,95±0,48**	**3,72±0,46**	**p=0,041**
**Tilburgs et al. 2020**	Diskutierte ACP-Präferenzen	6 Monate	**2,3±2,99**	**0,2±0,7**	**p<0,001**
	Diskutierte medizinische Präferenzen	6 Monate	**0,8±1,2**	**0,1±0,5**	**p=0,003**
	Diskutierte nicht-medizinische Präferenzen	6 Monate	**1,5±2,1**	**0,1±0,4**	**p<0,001**
	Lebensqualität (DEMQOL)	6 Monate	58,1±6,9	57,8±7,1	p=0,8
	Erlebtes SDM-Level	6 Monate	21,7±2,9	22,1±3,4	p=0,53
	Erlebtes Kompetenzlevel (SCQ)	6 Monate	94,3±11,9	97,8±13,3	p=0,17
	QALYs	6 Monate	0,3±0,1	0,3±0,1	p=0,43
	Gesundheitskosten (RUD)	6 Monate	10111,7±4505,6	10412,6±3344,7	p=0,66

Abkürzungen: ACP: Advance Care Planning; IG: Interventionsgruppe; KG: Kontrollgruppe; QALYs: qualitätskorrigierte Lebensjahre; SDM: Shared Decision-Making

## Diskussion

Im Rahmen des systematischen Reviews konnten sieben RCTs zu Auswirkungen von ACP auf MmD und ihre Pflegenden identifiziert und analysiert werden. Alle eingeschlossenen Studien konnten signifikanten Verbesserungen für MmD und deren Pflegende in unterschiedlichen Domänen nachweisen. Insgesamt erlaubten die Ergebnisse der identifizierten RCTs jedoch keine eindeutige Beurteilung der Wirksamkeit der ACP-Interventionen. Dies lag unter anderem an der unterschiedlichen Methodik und Qualität der durchgeführten Studien. Des Weiteren erschwerte die Heterogenität der erhobenen Outcomes einen direkten Vergleich der Interventionen und ließ nur bedingt eine Gesamtinterpretation der Ergebnisse zu.

Darüber hinaus hat sich die Mehrheit der eingeschlossenen Studien auf die Messung von Prozessparametern sowie von Endpunkten aus dem Bereich Kommunikation, Symptommanagement oder Lebensqualität konzentriert. Wichtige Ergebnisparameter von ACP blieben dagegen in vielen Studien unberücksichtigt. Insbesondere die Übereinstimmung der Wünsche der Betroffenen mit der tatsächlich durchgeführten Behandlung, das postulierte Ziel von ACP, wird in keiner der analysierten Studien erfasst. Wenngleich die Messung dieser Zielkonkordanz komplexe Herausforderungen mit sich bringt, erklärte auch eine internationale Delphi-Runde diese als wichtigsten Outcomeparameter von ACP-Interventionen 29 . Die im Rahmen dieser und vorangehender Arbeiten 10 17 18 19 30 festgestellte Diskrepanz zwischen den gemessenen Endpunkten und dem zentralen Ziel von ACP sowie die generelle Heterogenität der gemessenen Outcomes unterstreicht deutlich die Notwendigkeit, in künftigen Studien die Messung der Zielkonkordanz zu verbessern und die Outcomes weiter zu standardisieren. Des Weiteren zeigte die Synthese der Studien, dass die MmD in den berücksichtigten Studien nach wie vor nur in geringem Maße direkt in ACP-Interventionen miteinbezogenen wurden und die Mehrheit der Interventionen erst in fortgeschrittenen Demenzstadien einsetzten. Bereits Kelly et al. 19 stellten in ihrer Übersichtsarbeit fest, dass das Ausmaß der Teilhabe von MmD stark vom Setting abhängt. Die Wahrscheinlichkeit, dass MmD bei Interventionen im häuslichen Umfeld miteinbezogen werden, sei demnach deutlich höher als bei Personen, die bereits im Pflegeheim lebten. Die Mehrheit der Interventionen, der im vorliegenden Review eingeschlossenen Studien, wurde in Pflegeheimen durchgeführt 23 25 26 27 . Zudem befand sich der Großteil der Studienteilnehmenden bereits in weiter fortgeschrittenen Demenzstadien 23 24 26 . Das Kernziel von ACP ist es die individuellen Präferenzen der Betroffenen im klinischen Handeln zu berücksichtigen und eine patientenzentrierte Versorgung zu gewährleisten. Um MmD in ACP-Prozesse bestmöglich einzubinden, wären daher aufgrund der Progredienz der Erkrankung, möglichst früheinsetzende ACP-Interventionen geboten. Bislang wurden solche Modelle aber nur in wenigen Primärstudien untersucht 31 32 und die Aussagekraft dieser Studien ist aufgrund der Qualität und des gewählten Studiendesigns beschränkt. Hier wären rigorose randomisiert-kontrollierte Studien wünschenswert.

Neben der mangelnden frühzeitigen Einbindung von MmD in ACP bestehen weitere demenzspezifische Barrieren für eine erfolgreiche Implementation. Demnach spielen insbesondere Unsicherheiten hinsichtlich des Timings und der verschiedenen ACP-Optionen sowie mangelndes Wissen über Demenz und den Krankheitsverlauf eine entscheidende Rolle 33 34 35 . Für Allgemeinmediziner bestehen darüber hinaus oftmals Schwierigkeiten in der Beurteilung der Entscheidungsfähigkeit von MmD und im Umgang mit sich verändernden Präferenzen 34 .

Zusammenfassend unterstreichen die Ergebnisse des systematischen Reviews, dass ACP-Interventionen, wie Videos und Schulungen, ein wirksames Instrument sein können, um ACP für MmD und ihre Pflegenden erfolgreich umzusetzen. Es wird jedoch auch deutlich, dass die derzeitige Evidenz nicht ausreicht um die Wirksamkeit von ACP und die Überlegenheit von bestimmten Interventionen gegenüber anderen abschließend zu beurteilen. Es ist daher dringend notwendig die Wirksamkeit von komplexen ACP-Interventionen im Rahmen von qualitativ hochwertigen Studien zu untersuchen. Dabei gilt es sowohl die verwendeten Outcomes zu standardisieren als auch die Wirksamkeit der Interventionen hinsichtlich der primären Zielsetzung von ACP, und nicht nur in Bezug auf Prozessparameter, zu evaluieren.

## Limitationen

Aufgrund der Heterogenität der Definitionen von ACP und der Einschränkungen hinsichtlich Studiendesign, Publikationsjahr und Sprache ist nicht auszuschließen, dass relevante Artikel nicht identifiziert wurden und deshalb im Rahmen der Synthese nicht berücksichtigt wurden.

## Notes

FAZIT Die Ergebnisse der systematischen Übersichtsarbeit zeigen, dass ACP das Potential hat die Behandlungsplanung und Versorgungssituation von MmD und ihre pA signifikant zu verbessern. Für die Gewährleistung einer personenzentrierten und bedürfnisorientierten Versorgung von MmD, ist eine frühzeitige und systematische Implementierung von ACP unter Berücksichtigung möglicher Barrieren essenziell. Um ACP gewinnbringend zu realisieren, ist daher die Förderung von Schulungen und Aufklärung zum Thema ACP bei MmD unerlässlich.

## Zusätzliches Material

Zusätzliches Material Zusätzliches Material
