# Supplementary material for: Evidence on Advance Care Planning for People with Dementia – A Systematic Review (2017–2023)
Source: Gesundheitswesen. 2025 Nov 27;88(1):40–50. [Article in German] doi: 10.1055/a-2699-8545 (PMC12815580; doi:10.1055/a-2699-8545)
Supplement: Supplementary file 1 — Zusätzliches Material [file 10-1055-a-2699-8545-gesu-2024-08-2121-ua.pdf]

Thieme

Online Supplement

Online Supplement 1: Datenbanken und Suchstrategie

Datenbank	Suchstrategie	Jahre	Filter	Treffer
MEDLINE	((dementia OR dement* OR alzheim* OR frontotemporal dementia OR lewy body disease) AND (advance care planning OR advance* care plan* OR advance* directive* OR advance* decision* OR end of life decision* OR end-of-life-decision* OR healthcare directive* OR healthcare proxy OR future care plan OR terminal care plan* OR palliative care plan* OR shared decision making OR "living will"))	2017-2023	keine	2.100
Scopus	((dementia OR dement* OR alzheim* OR "frontotemporal dementia" OR "lewy body disease") AND ("advance care planning" OR "advance* care plan*" OR "advance* directive*" OR "advance* decision*" OR "end of life decision*" OR "end-of-life-decision*" OR "healthcare directive*" OR "healthcare proxy" OR "future care plan" OR "terminal care plan*" OR "palliative care plan*" OR "shared decision making" OR "living will"))	2017-2023	(LIMITTO (PUBSTAGE , "final")) AND (LIMITTO (DOCTYPE , "ar")) AND (LIMITTO (LANGUAGE , "English") OR LIMITTO (LANGUAGE , "German")) AND (LIMITTO (SRCTYPE , "j") OR LIMIT-TO (SRCTYPE , "d"))	4.816
CINAHL	((dementia OR dement* OR alzheim* OR frontotemporal dementia OR lewy body disease) AND (advance care planning OR advance* care plan* OR advance* directive* OR advance* decision* OR end of life decision* OR end-of-life-decision* OR healthcare directive* OR healthcare proxy OR future care plan OR terminal care plan* OR palliative care plan* OR shared decision making OR "living will"))	2017-2023	Expanders - Apply equivalent subjects<br>Search modes - Boolean/Phrase	718
CENTRAL	((dementia OR dement* OR alzheim* OR frontotemporal dementia OR lewy body disease) AND (advance care planning OR advance* care plan* OR advance* directive* OR advance* decision* OR end of life decision* OR end-of-life-decision* OR healthcare directive* OR healthcare proxy OR future care plan OR terminal care plan* OR palliative care plan* OR shared decision making OR "living will")) (Word variations have been searched)	2017-2023	Word variations have been searched	691
PsycInfo	((dementia OR dement* OR alzheim* OR frontotemporal dementia OR lewy body disease) AND (advance care planning OR advance* care plan* OR advance* directive* OR advance* decision* OR end of life decision* OR end-of-life-decision* OR healthcare directive* OR healthcare proxy OR future care plan OR terminal care plan* OR palliative care plan* OR shared decision making OR "living will"))	2017-2023	Expanders - Apply equivalent subjects<br>Search modes - Boolean/Phrase	327
**Summe**				**8.652**

Online Supplement 2: Ein- und Ausschlusskriterien

	**Einschlusskriterien**	**Ausschlusskriterien**
**Population**	• Erwachsene Menschen mit einer primären Demenzform<br>• Pflegende An- und Zugehörige von Menschen mit Demenz<br>• Medizinisches Fachpersonal für Menschen mit Demenz (z.B. Pflegekräfte, Ärzte)	• Menschen mit/Angehörige von Menschen mit Formen kindlicher Demenzen (z.B. Neuronale Ceroid-Lipofuszinosen)<br>• Menschen mit/Angehörige von Menschen mit anderen terminalen Erkrankungen (z.B. chronische Nierenerkrankung, Krebserkrankungen)
**Intervention**	• ACP	• Nicht ACP
**Vergleichsgruppe**	• Standardversorgung, keine Intervention<br>• alternative Intervention ohne ACP	• Vergleichsintervention mit ACP-Elementen
**Zielgröße**	• alle Endpunkte, die die Wirksamkeit von ACP erfassen	• Endpunkte, die nicht die Wirksamkeit von ACP erfassen
**Studiendesign**	• Randomisiert kontrollierte Studien	• Studiendesigns ohne Kontrollgruppe und/oder randomisierte Gruppenzuteilung<br>• Studienprotokolle
**Sprache**	• Englisch und Deutsch	• Volltexte in nicht englischer oder deutscher Sprache
**Zeitraum**	• 2017-2023	• < 2017
